# RNA sequencing analysis reveals quiescent microglia isolation methods from postnatal mouse brains and limitations of BV2 cells

**DOI:** 10.1186/s12974-018-1195-4

**Published:** 2018-05-22

**Authors:** Yingbo He, Xiang Yao, Natalie Taylor, Yuchen Bai, Timothy Lovenberg, Anindya Bhattacharya

**Affiliations:** 1 Janssen Research & Development, LLC., Neuroscience Drug Discovery, 3210 Merryfield Row, San Diego, CA 92121 USA; 2 Janssen Research & Development, LLC., Discovery Sciences, San Diego, CA USA; 3 Janssen Research & Development, LLC., Discovery Sciences, Spring House, PA USA

**Keywords:** Primary microglia, BV2, RNA sequencing, Transcriptome, Function

## Abstract

**Background:**

Microglia play key roles in neuron–glia interaction, neuroinflammation, neural repair, and neurotoxicity. Currently, various microglial in vitro models including primary microglia derived from distinct isolation methods and immortalized microglial cell lines are extensively used. However, the diversity of these existing models raises difficulty in parallel comparison across studies since microglia are sensitive to environmental changes, and thus, different models are likely to show widely varied responses to the same stimuli. To better understand the involvement of microglia in pathophysiological situations, it is critical to establish a reliable microglial model system.

**Methods:**

With postnatal mouse brains, we isolated microglia using three general methods including shaking, mild trypsinization, and CD11b magnetic-associated cell sorting (MACS) and applied RNA sequencing to compare transcriptomes of the isolated cells. Additionally, we generated a genome-wide dataset by RNA sequencing of immortalized BV2 microglial cell line to compare with primary microglia. Furthermore, based on the outcomes of transcriptional analysis, we compared cellular functions between primary microglia and BV2 cells including immune responses to LPS by quantitative RT-PCR and Luminex Multiplex Assay, TGFβ signaling probed by Western blot, and direct migration by chemotaxis assay.

**Results:**

We found that although the yield and purity of microglia were comparable among the three isolation methods, mild trypsinization drove microglia in a relatively active state, evidenced by high amount of amoeboid microglia, enhanced expression of microglial activation genes, and suppression of microglial quiescent genes. In contrast, CD11b MACS was the most reliable and consistent method, and microglia isolated by this method maintained a relatively resting state. Transcriptional and functional analyses revealed that as compared to primary microglia, BV2 cells remain most of the immune functions such as responses to LPS but showed limited TGFβ signaling and chemotaxis upon chemoattractant C5a.

**Conclusions:**

Collectively, we determined the optimal isolation methods for quiescent microglia and characterized the limitations of BV2 cells as an alternative of primary microglia. Considering transcriptional and functional differences, caution should be taken when extrapolating data from various microglial models. In addition, our RNA sequencing database serves as a valuable resource to provide novel insights for appropriate application of microglia as in vitro models.

**Electronic supplementary material:**

The online version of this article (10.1186/s12974-018-1195-4) contains supplementary material, which is available to authorized users.

## Background

Microglia are essential components of the central nervous system (CNS) with a broad range of roles in neurodevelopment, homeostasis, synaptic plasticity, and injury responses [1–3]. In healthy brain, microglia survey the brain parenchyma dynamically [4]. During pathological conditions, microglia dysfunction contributes to the pathogenesis of most neurodegenerative diseases and psychological disorders [5]. To define the mechanisms underlying microglia function and to investigate the potential utility of these cells as druggable targets, it is indispensable to establish reliable in vitro models for microglia.

Primary microglia are a useful in vitro model for mechanistic studies and compound testing because they recapitulate a majority of known physiological activities of microglia in vivo, including phagocytosis, migration, and release of pro-inflammatory cytokines and chemokines when stimulated [6]. For now, however, the major research works of microglia, particularly those on fundamental transcriptome and proteome profiles, were obtained based on primary microglia derived from different isolation methods. For instance, microglia isolated with CD11b magnetic bead sorting (MACS) were used for whole genome analysis among different brain regions [7]. Microglia proteomic identification [8] and transcriptome signature upon lipopolysaccharide (LPS) [9] were based on microglia isolated by the shaking method. Moreover, transcriptional changes of microglia isolated from mild-trypsin digestion were investigated in mouse EAE model [10]. Despite lots of progress, difficulties exist for parallel comparison between studies with microglia from distinct isolation methods because microglia are sensitive and likely to be activated to a certain extent during each isolation procedure [11]. Furthermore, microglia activation may mask the differences across interventions. Therefore, establishing a reliable quiescent microglia model is vital, which can be potentially used for comparing gene expression and functions in response to different treatments. Due to the requirement of high yield and further cellular culture for mechanic investigation and drug discovery, we isolated microglia from P0–P3 postnatal mouse brains with a mixed glial culture system. The three most popular microglial isolation methods from the mixed glial culture system are shaking, mild trypsinization, and CD11b MACS. In previous literature, although the yield, purity, and viability of microglia have been compared [12, 13], no one has ever explored the whole transcriptional level of microglia across different isolation approaches. Hence, one of our aims here is to conduct such a comparison and find out the best approach for in vitro microglial isolation and culture from postnatal mouse brains, which is, we believe, of great value to guide future studies.

In addition to primary microglia, microglia-like cell lines have been created and extensively used for examining mechanistic details of microglial function, which include mouse immortalized BV2 cells. Although immortalized cells replicate readily and are easy to maintain in culture, their validity as a sufficient substitute for primary microglia has been debated [14]. Functionally, microglia cell lines share similarities with primary microglia but are separated by crucial differences in secretion as well as gene expression upon LPS stimulation [15, 16]. Although distinct expression patterns of specific microglial genes have been reported between BV2 cells and mouse primary microglia [17], the whole transcriptome signature and their functional differences still need to be further explored.

To this end, we compared transcriptome of isolated primary microglia across the three different isolation methods (shaking, mild trypsinization, and CD11b MACS), as well as with BV2 cells. According to their distinct transcriptome profiles and pathway enrichment, we then performed in-parallel functional assays between primary microglia and BV2 cells regarding LPS responses, transforming growth factor beta (TGFβ) signaling, and chemotaxis. Taken together, we determined the optimal isolation methods for quiescent microglia; transcriptional and functional analyses also revealed that BV2 cells may not adequately represent primary microglia, which offered valuable insights into the selection of appropriate microglial in vitro models under certain circumstances.

## Methods

### Animals

Wild-type C57BL/6 pregnant mice were obtained from Charles River Laboratories, Inc. Mice were allowed to acclimate for 7 days after receipt. They were kept on a 12-h light/dark cycle and allowed free access to food and water. All animal care and use were in accordance with the Institutional Animal Care and Use Committee (IACUC)-approved protocols.

### Mouse microglia isolation and culture

Cortices from P0–P3 C57BL/6 mouse pups were dissected and stripped of meninges and mechanically dissociated with a hand homogenizer and a 25-gauge needle. The cell suspension was seeded into poly-l-lysine-coated (Sigma-Aldrich) T150 tissue culture flasks and maintained in DMEM/F12 with 10% FBS and 1% penicillin–streptomycin for 10–14 days to grow a confluent mixed astrocyte/microglia population.

### Shaking purification

The confluent mixed glia cultures were shaken in an orbital shaker at 37 °C with 230 rpm for 2 h. The floating cells were collected, centrifuged, and plated on a poly-l-lysine-coated plate for 24 h. Cultures were washed twice to remove cell debris before RNA was extracted using RNeasy Plus Mini Kit (QIAGEN).

### Mild-trypsin purification

Mild trypsinization was performed as described before [13]. Briefly, trypsin–EDTA solution (0.25% trypsin, cat. no. 25200-072; Invitrogen) diluted 1:4 in PBS containing Ca^2+^ was applied to the mixed glial cells. The upper mixed glial cell layer slowly became detached from the bottom of the flask after incubation with this mild trypsin for at least 30 min depending on the culture confluence at 37 °C. When the top layer of mixed glial cells became completely detached, trypsin was inhibited by adding 10% of serum to the flask and the detached upper cells and the trypsin solution were discarded. All that were left at the bottom of the flask were adherent microglial cells. The cells were either directly scraped for RNA extraction or re-plated into a poly-l-lysine-coated plate for 24 h before RNA extraction using RNeasy Plus Mini Kit (QIAGEN).

### CD11b MACS purification

We gently scraped and applied the cells to an antigen–antibody-mediated magnetic cell-sorting (MACS, Miltenyi Biotec) assay to positively select microglia. Briefly, the mixed glial population was re-suspended in MACS buffer (Miltenyi Biotec) and incubated with CD11b MicroBeads (Miltenyi Biotec). The cell suspension was then applied to LS separation column (Miltenyi Biotec) fitted into a QuadroMACS cell separator (Miltenyi Biotec). Unlabeled cells were allowed to pass through the column while labeled cells remained captured in the magnetic field. After washing the column with MACS buffer, the column was then removed from the magnetic separator and flushed with MACS buffer to collect the purified microglia population. For an increased level of purity, the eluted microglia population was passed through a new LS separation column a second time. The purity of microglia used in our study was more than 95% assessed by immunocytochemistry (data not shown). Microglia either acutely collected from the LS separation column or incubated on a poly-l-lysine-coated plate for 24 h were homogenized, and total RNA was extracted using RNeasy Plus Mini Kit (QIAGEN).

### BV2 cell line

The microglia BV2 cell line was obtained from Dr. Dennis Selkoe (Harvard University) and cultured in DMEM with 10% FBS and 1% penicillin–streptomycin.

### Immunocytochemistry

Immunocytochemistry was performed as described previously. Briefly, cells were fixed with 4% paraformaldehyde and permeabilized by 0.1% Triton X-100. After blocking with 10% donkey serum, fixed cells were incubated with primary antibodies (Iba1, 1:1000, WAKO Chemicals; GFAP, 1:1000, Abcam) for 2 h followed by fluorochrome-conjugated secondary antibodies (Alexa Fluor 488, Alexa Fluor 555, 1:200, Molecular Probes, respectively). Nuclei were counterstained with DAPI. Fluorescence images were acquired using a confocal-laser microscope (LSM 700; Carl Zeiss MicroImaging) with a multi-track configuration.

### Microglial purity and morphological analysis

The purity of isolated microglia with each isolation method was determined by the percentage of Iba1^+^ cells in total cells, which was indicated by immunocytochemical staining using DAPI and the antibodies against Iba1 and GFAP. For morphological analysis, we defined amoeboid microglia as flat Iba1^+^ cells without thin processes and calculated the percentage of amoeboid microglia in total microglial cells. At least five randomly selected fields were used for quantification.

### RNA sequencing and data processing

RNA quality was assessed by using Agilent RNA 6000 Nano Kit and Agilent 2100 Bioanalyzer according to the manufacturer’s instructions. Qualified total RNA (RIN > 9, 200 ng) from each sample was processed by following TruSeq RNA Library Prep Kit v2 protocol (Illumina, San Diego, CA). In brief, poly-A containing mRNA purified from each total RNA samples was applied to cDNA library construction. The libraries were sequenced at pair end with read length of 100 bp on Illumina HiSeq 4000 platform at a depth of more than 40 million reads. The experiments were carried out by BGI Americas (Cambridge, MA), a fee-for-service provider.

ArrayStudio version 8.0 (Omicsoft, Cary, NC) was applied to quality control (QC) raw RNA sequencing reads, map reads to genome, quantify gene expression, and test expression changes. In brief, low-quality bases and adaptors were trimmed and reads less than 25 bases were discarded. Remaining reads were mapped to mouse GRCm38 genomes (https://www.ncbi.nlm.nih.gov/grc/mouse) using Omicsoft sequence aligner (OSA) [18] of the ArrayStudio software. Gene expression read count and TPM (Transcript Per kilobase Million) were calculated based on mouse version m10 of GenCode gene models (https://www.gencodegenes.org/mouse_releases/10.html/). Samples in each group were QCed-based overall gene expression consistency, and outliers were removed before downstream analysis. Robust center scale was applied to normalize data in all heat maps.

We deposited raw read fastq and sample metadata files in NCBI with BioProject ID PRJNA407656.

### Differential gene expression and pathway enrichment analysis

Inference tests based on the Voom algorithm [19] were applied to adjust read depth differences between samples and estimate changes or differences of gene expression when comparing sample groups. Genes with little or no expression (average TPM < 0.1) were excluded from inference tests. Differentially expressed genes (DEG) from the inference test were selected according to expression changes of more than fourfold and adjusted *P* value (calculated by Benjamini–Hochberg procedure) of less than 0.05, or stated otherwise.

MetaCore database version 6.31 (https://clarivate.com/products/metacore/) was applied to analyze the enrichment of DEGs in biological pathways and processes. Enrichment of significant pathways (adjusted *P* value < 0.05, calculated by the database) in each analysis was exported from the database and charted using ArrayStudio version 8.0 or Excel.

### Integration of published data

Raw microarray data of published studies on microglia cells with LPS treatment (GSE49329), beta amyloid peptide treatment (GSE55627), and aging (GSE62420) were retrieved from GEO (https://www.ncbi.nlm.nih.gov/geo/). Custom CDF (ENTREZG version 18, http://brainarray.mbni.med.umich.edu/www/data-analysis/custom-cdf/) was applied to extract gene expression data from raw CEL files, and standard inference tests were applied in treated versus control comparisons. Genes in treatment groups with expression level significantly (adjusted *P* value (calculated by Benjamini–Hochberg procedure) < 0.05) induced more than twofold compared with that in control groups in each study were collected for further analysis.

### Quantitative real-time PCR

RNA was reverse-transcribed into cDNA using Superscript III Reverse Transcriptase (Invitrogen) with random hexamer primers. Transcript abundance was determined by quantitative PCR using SYBR Green PCR Mix (Applied Biosystems) with the following primer pairs:

*Tspo*: GCCTACTTTGTACGTGGCGAG (F), CCTCCCAGCTCTTTCCAGAC (R);

*Ptgs2*: TTCAACACACTCTATCACTGGC (F), AGAAGCGTTTGCGGTACTCAT (R);

*Cd86*: TGTTTCCGTGGAGACGCAAG (F), TTGAGCCTTTGTAAATGGGCA (R);

*Tnfa*: CCCTCACACTCAGATCATCTTCT (F), GCTACGACGTGGGCTACAG (R);

*Il6*: TAGTCCTTCCTACCCCAATTTCC (F), TTGGTCCTTAGCCACTCCTTC (R);

*Il1b*: GCAACTGTTCCTGAACTCAACT (F), ATCTTTTGGGGTCCGTCAACT (R);

*Tgfb1*: CTCCCGTGGCTTCTAGTGC (F), GCCTTAGTTTGGACAGGATCTG (R);

*Tgfbr1*: TCTGCATTGCACTTATGCTGA (F), AAAGGGCGATCTAGTGATGGA (R);

*Tgfbr2*: CCGCTGCATATCGTCCTGTG (F), AGTGGATGGATGGTCCTATTACA (R);

*Serpine1*: TTCAGCCCTTGCTTGCCTC (F), ACACTTTTACTCCGAAGTCGGT (R);

*C5a*: GAACAAACCTACGTCATTTCAGC (F), GTCAACAGTGCCGCGTTTT (R);

*C5ar1*: TACCATTAGTGCCGACCGTTT (F), CCGGTACACGAAGGATGGAAT (R);

*C5ar2*: CTGCTGTCTACCGTAGGCTG (F), AGAGGAATCGAACAGTGGTGA (R);

*Gapdh*: AGGTCGGTGTGAACGGATTTG (F), TGTAGACCATGTAGTTGAGGTCA (R).

### Secretome analysis

Secretome assay was carried out as described before [20]. Briefly, the relative concentrations of secreted proteins in cell supernatants were measured using antibody-based 38-plex immunoassays (Luminex, R&D systems). The 38 secreted proteins were the following: CCL2/JE/MCP1, CCL3/MIP1α, CCL4/MIP1β, CCL5/RANTES, CCL20/MIP3α, CXCL1/KC, CXCL2/MIP2, CXCL10/IP10/CRG2, CXCL12/SDF1α, FGFb, FGF21, GCSF, GMCSF, IFNγ, IGFI, IL1α, IL1β, IL2, IL4, IL5, IL6, IL10, IL12 p70, IL13, IL17A, IL23 p19, IL33, LIX, MCSF, MMP9, Resistin, TNFα, VEGF, CCL11/Eotaxin, CCL22/MDC, CXCL9/MIG, IL9, and RAGE. We then normalized immunoassay measurements of the listed proteins and clustered them using an unsupervised clustering algorithm (Array Studio) to generate proteomic heat maps. Any undetectable proteins for a sample were removed from the analysis.

### Western blot

Cells were homogenized and lysed using RIPA buffer (Amresco) with protease and phosphatase inhibitors (Sigma and Roche, respectively). After centrifugation at 13,000*g* for 5 min, protein concentrations were measured using the BCA protein assay kit (Pierce) and lysates were separated on a 4–12% Bis–Tris gels (Invitrogen) using MOPS sodium dodecyl sulfate running buffer (Invitrogen). Proteins were transferred with the iBlot system onto nitrocellulose membranes (Novex) and incubated with antibodies p-Smad2 (1:1000, Millipore) and Smad2 (1:1000, Cell Signaling Technology). Signal intensities were detected using ECL Western blotting detection reagents (Amersham Biosciences) and evaluated by ImageJ.

### Chemotaxis

Cells were seeded into the upper chamber of an ICAM-precoated separate culture plate inserts (Sartorius) with DMEM/F12 containing 0.5% FBS. The same culture medium and 11 nM C5a were added to the lower chamber. Chemotaxis was monitored every hour for 72 h by IncuCyte Zoom live-cell system (Sartorius).

### Statistical analysis

Data were statistically compared using *t* test between two groups, one-way ANOVA followed by Tukey’s post hoc test among multiple groups, and two-way ANOVA among and within groups using GraphPad Prism 7 (GraphPad Software, Inc.). *P* < 0.05 was considered statistically significant.

## Results

### Characterization of isolated microglia

**Fig. 1:**
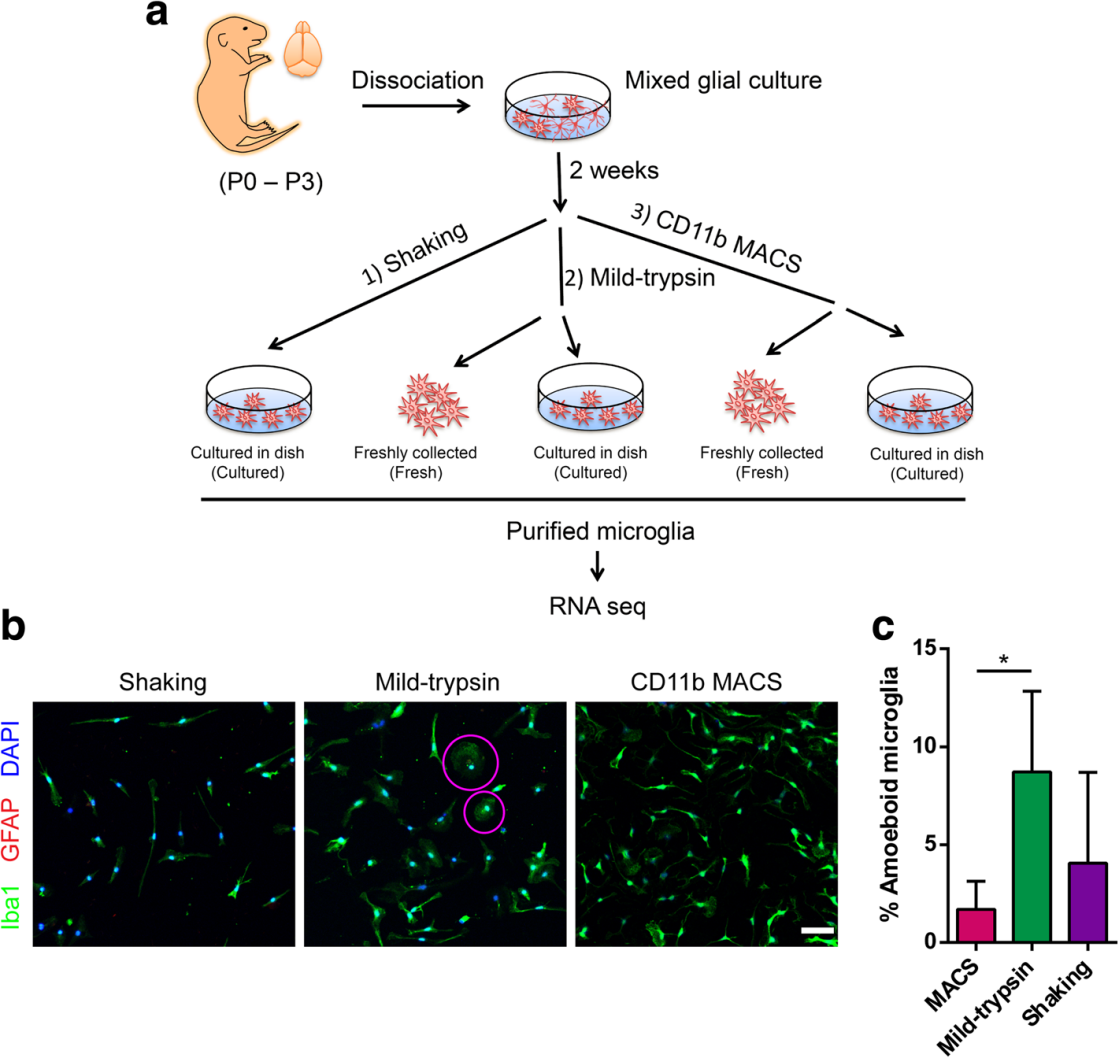
Characterization of microglia samples for RNA-Seq. **a** Scheme showing the workflow for isolation of mouse microglia with three different methods and samples we used for RNA-Seq analysis. **b** Representative images showing purity and morphology of isolated microglia by immunostaining (Iba1^+^, green; GFAP^+^, red). DAPI indicates nuclei. Purple circles highlight amoeboid microglia. Scale bar, 50 μm. **c** The histogram showing the percentage of amoeboid microglia induced by the three isolation methods. Data are shown as mean + SD, *n* = 5. One-way ANOVA followed by Tukey’s post hoc test. *P < 0.05

To compare different microglial isolation methods, we freshly collected or cultured microglia isolated from P0–P3 mouse brains by using shaking, mild trypsinization, and CD11b MACS after mixed glial cultures (Fig. 1a). It should be noted that we could not collect shaking-isolated fresh microglia because of high amounts of cell debris produced during the isolation process. Prior to RNA-Seq, cell purity was assessed by immunocytochemistry with the microglial marker Iba1 and the astrocyte marker GFAP. As shown in Fig. 1b and Additional file 1, the purity of microglia isolated with different methods was above 95% as indicated by minimal or no GFAP staining. Data also suggested that microglia obtained by different approaches exhibited varied cellular morphology, as indicated by Iba1 immunostaining (Fig. 1b). In particular, shaking- and CD11b MACS-isolated microglia displayed polarized processes and lamellipodium, while those obtained by mild trypsinization exhibited amoeboid morphology (Fig. 1b). According to this observation, we quantified the percentage of amoeboid microglia derived from the three isolation methods and found that the mild trypsinization method produced higher number of amoeboid microglia than the CD11b MACS method (Fig. 1c), implying greater activation of mild trypsinization-isolated microglia. For further analysis, we extracted total RNA of these isolated cells from four biological replicates and then performed RNA sequencing (RNA-Seq). In addition, BV2 cells were also subjected to RNA-Seq in parallel to characterize transcriptional differences between microglial cell line and primary microglia.

### RNA-Seq analysis of microglia

**Fig. 2:**
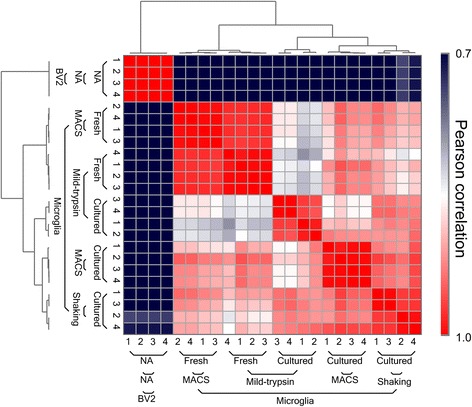
RNA-Seq analysis of isolated microglia. Heat map of pair-wise Pearson correlation between cell types with different isolation methods and biological independent replicates

With RNA-Seq, we obtained more than 40 million reads for each sample and more than 90% of all reads were uniquely mapped and paired (see Additional file 2). Results of Pearson correlation analysis using the whole transcriptome between all replicates of each cell type and isolation method indicated high data quality and consistent distinction among the cell groups (Fig. 2). The analysis clearly grouped microglia and BV2 cells into two clusters regardless of cell isolation methods, indicating that transcriptional differences exist between the two cell types and that BV-2 cells ought to be used with caution, as surrogates of microglia. Among all microglia samples, fresh and cultured cells were clustered separately regardless of isolation methods (Fig. 2), suggesting that culture process has greater impact on cells than the isolation methods per se. These were confirmed by principal component analysis (PCA) analysis (see Additional file 3). Further comparison revealed a stronger correlation between fresh and cultured cells isolated by CD11b MACS than that between fresh and cultured cells isolated by mild-trypsin digestion (Fig. 2), indicating that the transcriptome of microglia isolated by CD11b MACS has less changes under culture conditions. Altogether, these data confirmed the quality of the RNA-Seq analysis, which can be applied for further transcriptional characterization.

### Transcriptional changes of microglia by different isolation methods

**Fig. 3:**
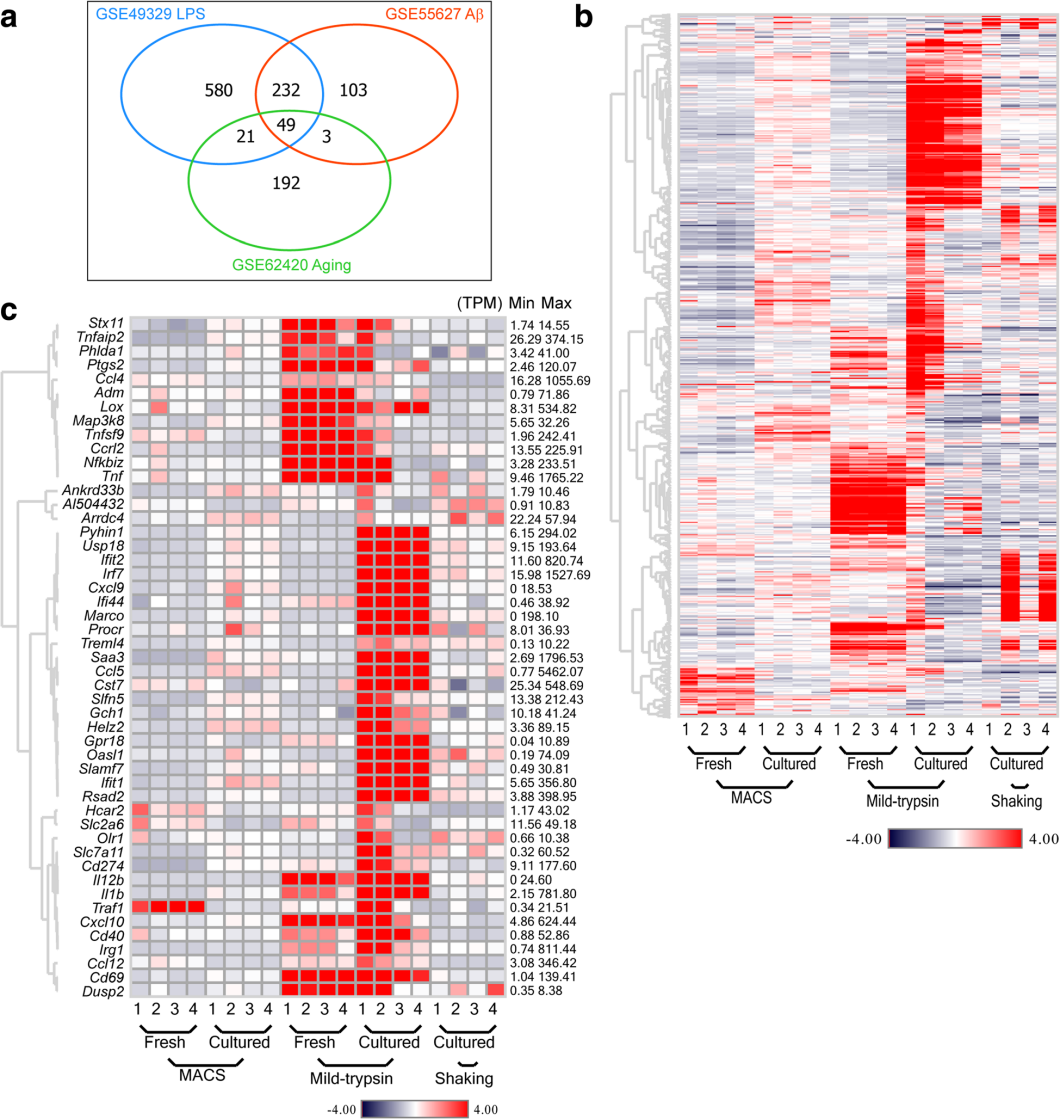
Different isolation methods stimulate microglial activation. Microglia were purified from the mixed glial system using CD11b MACS microbeads, shaking, or mild-trypsin digestion. **a** Venn diagram analyzed from publicly available microarray data with GEO series ID (GSE) showing similarities and differences of 1180 upregulated genes in microglia treated with LPS or Aβ or during aging. **b** Heat map and hierarchical clustering of all 1180 upregulated transcripts among microglia isolated by different methods. **c** Heat map and hierarchical clustering of the common 49 transcripts shared in **a** among microglia isolated by different methods. The minimal and maximal expression level of each transcript was indicated

Under physiological conditions, microglia constantly survey the brain environment with their ramified processes in a resting state [21]. Therefore, the isolated microglia in a minimal activation status will not only closely resemble the resting counterparts in vivo but also be a sensitive and reliable model for testing different treatments in vitro. To this end, we sought to determine which isolation method minimally activates microglia. In order to identify genes linked to microglial activation, we utilized published studies of activated microglia induced by LPS [22], Aβ [23], or aging [7] to screen reference genes. The top 1180 upregulated genes under each condition alone and the common 49 genes shared by all three conditions were selected as reference to measure the activation states of our microglia samples (Fig. 3a). Surprisingly, we found much higher expression of both 1180- and 49-gene panels in mild-trypsin-isolated microglia than in MACS- and shaking-isolated microglia (Fig. 3b, c), indicating that “more activated” microglia were isolated by mild trypsin. In addition, we listed fold changes and average expression levels of all transcripts in the cited studies and our study (see Additional files 4 and 5).

**Fig. 4:**
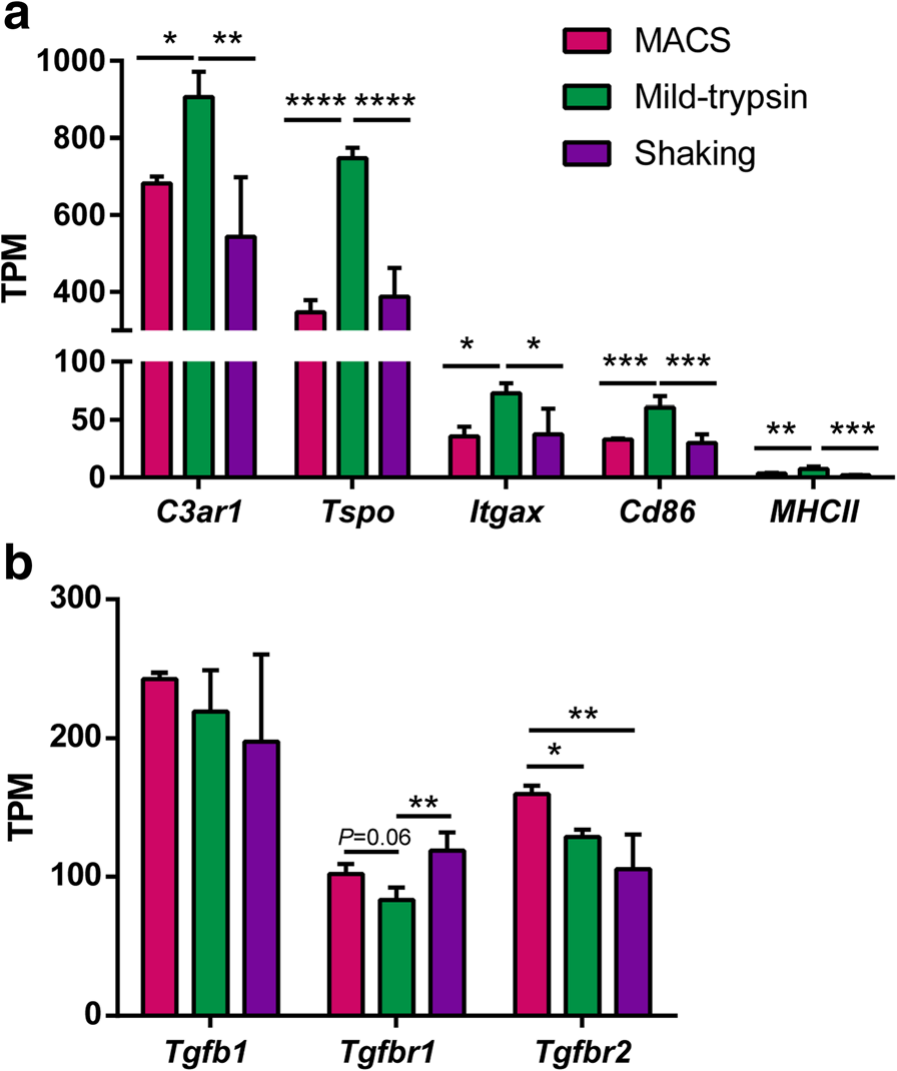
Transcriptional changes in isolated microglia from different isolation methods. **a** Expression of representative activation genes in microglia isolated by different methods. **b** Expression of representative quiescent genes in microglia isolated with different methods. Data are shown as mean + SD, *n* = 4. One-way ANOVA followed by Tukey’s post hoc test. *P < 0.05; **P < 0.01; ***P < 0.005; ****P < 0.0001

To further explore the transcriptional differences of isolated microglia derived from the three isolation methods, we examined the expression of common genes related to pro-inflammation. Consistently, we found that mild-trypsin-isolated microglia expressed significantly higher levels of general microglial activation genes than cells derived from the other two methods. The activation genes included *Tspo*, *C3ar1*, *Itgax*, *Cd86*, and *MHCII* (Fig. 4a). In contrast to microglial activation genes, TGFβ signaling is essential for microglia to maintain their quiescent states [24]. We then tested TGFβ signaling-related genes in isolated microglia among the three isolation methods. Although there were no changes in *Tgfb1* expression among differently isolated microglia, the levels of *Tgfbr1* and *Tgfbr2* were suppressed in mild-trypsin-isolated microglia (Fig. 4b), suggesting that mild trypsinization produced “less quiescent” microglia compared to other methods.

Altogether, the data demonstrate that mild-trypsin isolation method provided the highest level of microglial activation, which is consistent with the amoeboid morphology observed in this type of microglia (Fig. 1b, c). We thus used datasets from CD11b MACS- and shaking-isolated microglia as quiescent cell models for our further analysis.

### Transcriptome differences between quiescent primary microglia and BV2 cells

**Fig. 5:**
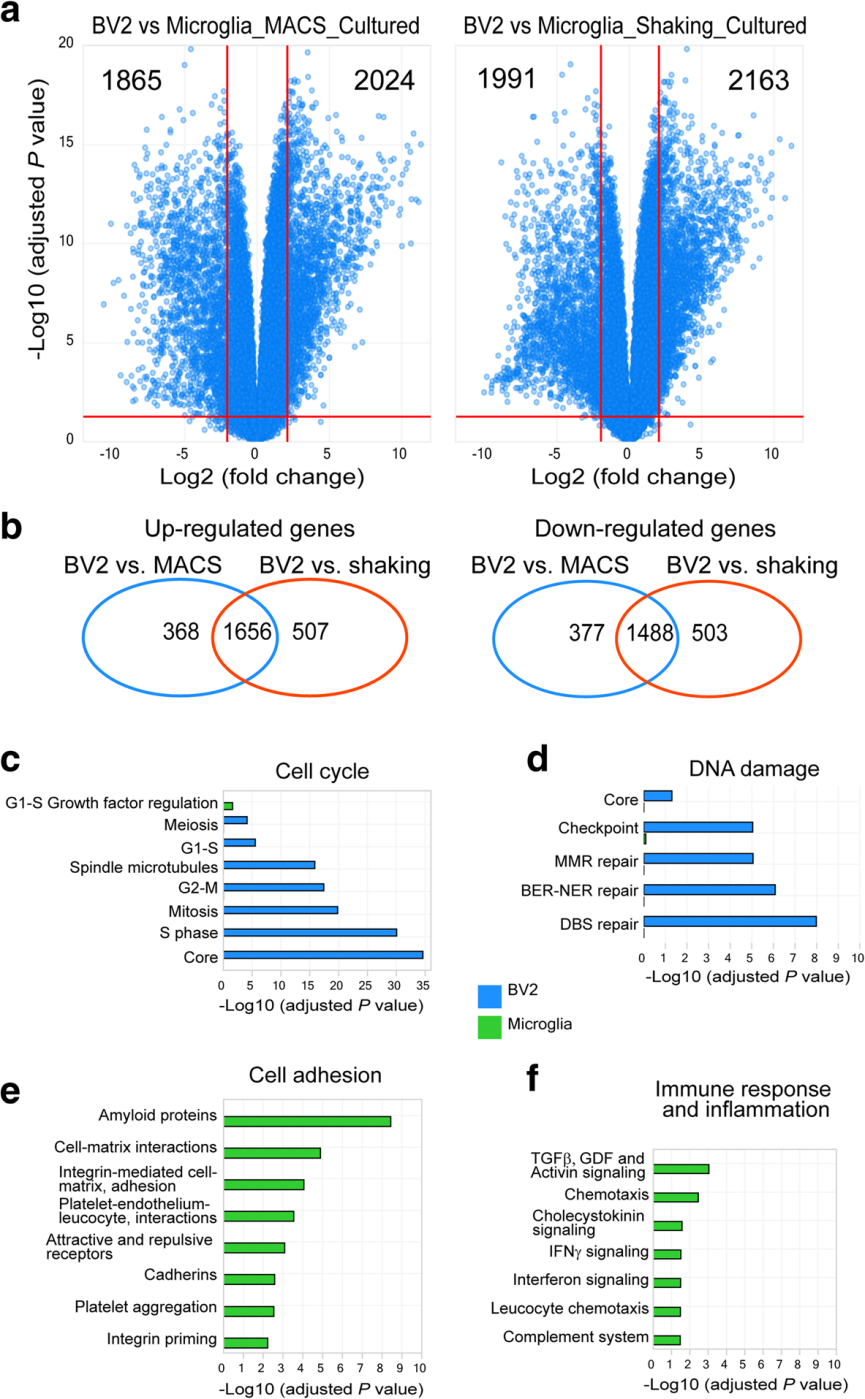
Transcriptional comparison of BV2 cells with primary microglia. **a** Volcano plots of genes showing the magnitude (log2 (fold change), *x*-axis) and significance (−log10 (adjusted *P* value), *y*-axis) for cultured BV2 cells compared to cultured microglia isolated with either MACS or shaking methods. Each spot represents a transcript. The horizontal red line indicates statistical significance threshold (adjusted *P* value < 0.05). Two vertical red lines show the threshold of fold changes (log2 (fold change) > 2 or <− 2). Number of significantly different transcripts was indicated in the corner. **b** Venn diagram showing similarities and differences of up- and downregulated transcripts of BV2 cells compared to MACS- or shaking-derived microglia analyzed in **a**. **c**–**f** Biological processes and pathways such as cell cycle (**c**), DNA damage (**d**), cell adhesion **(e)**, as well as immune response and inflammation (**f**), enriched in microglia (green) and BV2 cells (blue)

So far, we have documented that MACS- and shaking-isolated microglia are relatively quiescent. BV2 cells are an immortalized neonatal mouse microglial cell line, which is widely used as a surrogate of primary microglia in vitro [14]. To validate if BV2 cells can recapitulate most features of gene expression and biological function of primary microglia, we then compared the whole transcriptome between BV2 cells and quiescent MACS- and shaking-isolated primary microglia. We found 1656 genes with at least fourfold higher expression and 1488 genes with at least fourfold lower expression in BV2 cells than microglia shared by the two different isolation methods (Fig. 5a, b), providing insights into different transcriptional expression between BV2 cells and primary microglia. These highly abundant genes of BV2 cells were related to cell cycle and DNA damage (Fig. 5c, d), while those with low expression were related to cell adhesion (Fig. 5e). These findings were consistent with our observations that in culture plates, primary microglia adhered more strongly but proliferated much more slowly than BV2 cells and that DNA damage (evidenced by doublet nuclei) was usually discerned in a larger proportion of BV2 cells (data not shown). In addition, immune response and inflammation analysis based on the differentially expressed genes demonstrated that most microglia-specific immune functions and pathways were retained in BV2 cells except, but not limited to, TGFβ signaling and chemotaxis (Fig. 5f). Together, these results provide transcriptional evidences on the suitability of BV2 cells as a microglia surrogate when studying distinct functions and pathways.

### Responses of primary microglia and BV2 cells to LPS

**Fig. 6:**
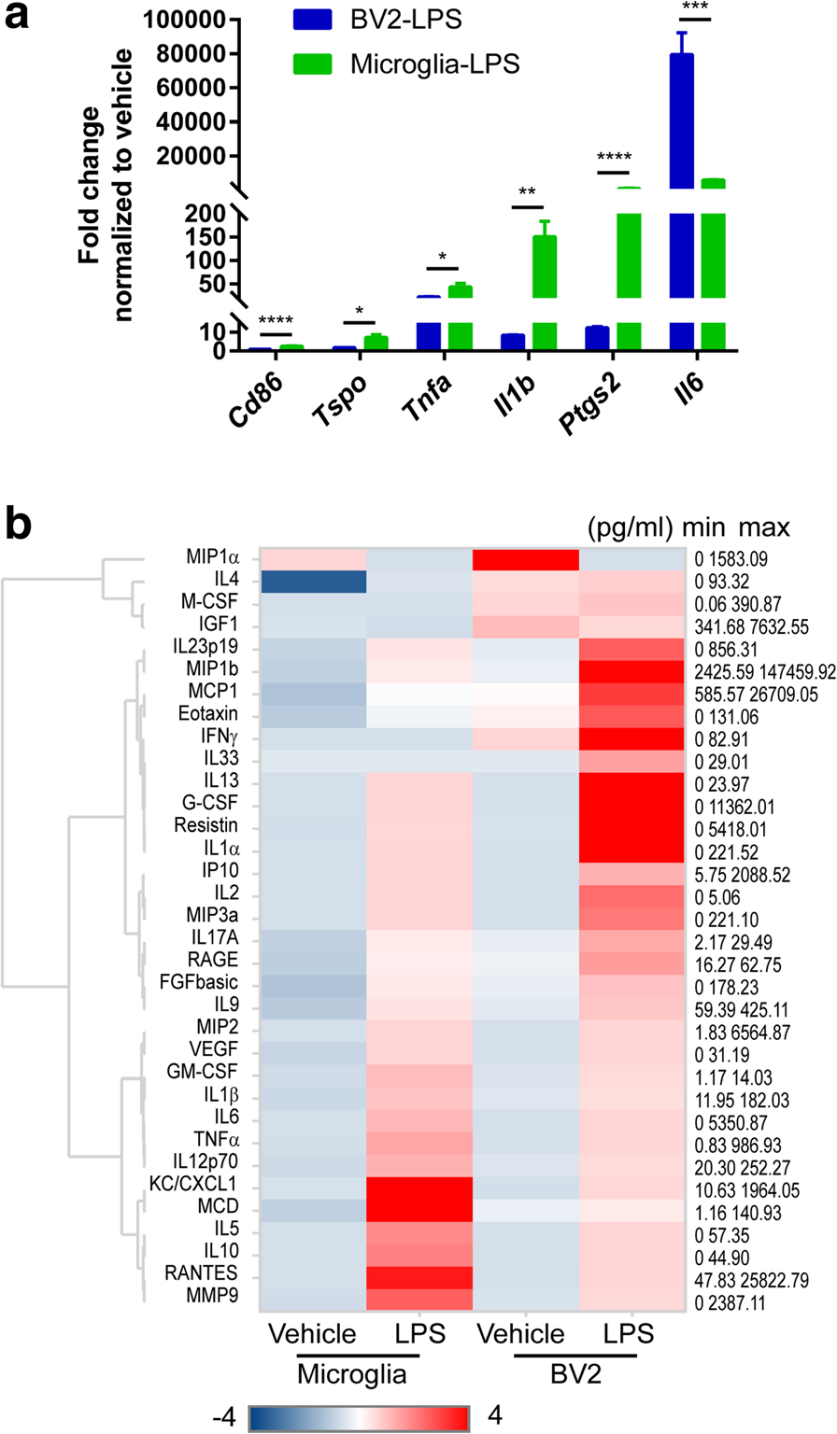
Immune responses of primary microglia and BV2 cells to LPS. Primary microglia isolated by the CD11b MACS method and BV2 cells were treated with 100 ng/ml LPS for 24 h. **a** Representative gene expression in primary microglia and BV2 cells measured by quantitative RT-PCR. Expression level was normalized to respective vehicle control. **b** Heat map and hierarchical clustering of detectable 34 proteins released from primary microglia and BV2 cells measured by Luminex Multiplex. The minimal and maximal secretion level of each protein was indicated. Data are shown as mean + SD of triplicates. Experiment repeated twice independently. Unpaired *t* test. *P < 0.05; **P < 0.01; ***P < 0.001; ****P < 0.0001

To validate the findings of RNA-Seq, we compared the functions between primary microglia and BV2 cells. We used MACS-derived primary microglia because the cells were quiescent and the method was consistent and repeatable. To confirm that BV2 cells and isolated primary microglia maintain functional characteristics of microglia, we assessed the immune responses of the two cell types to LPS, a pro-inflammatory agent used for neuroinflammation. We treated the cells with 100 ng/ml LPS for 24 h, then measured the pro-inflammatory genes and secreted proteins. Quantitative RT-PCR results showed that both types of the cells responded strongly to LPS, manifested by increased expression of *Cd86*, *Tspo*, *Tnfa*, *Il1b*, *Ptgs2*, and *Il6* (Fig. 6a). However, primary microglia exhibited higher levels of *Cd86*, *Tspo*, *Tnfa*, *Il1b*, and *Ptgs2* than BV2 cells except for *Il6* (Fig. 6a). We next tested the secretion of inflammation-related factors following LPS treatment. Interestingly, BV2 cells released slightly higher amounts of some proteins in our selected panel than primary microglia even at basal level (Fig. 6b), indicating that BV2 cells were in a more activated state. In response to LPS, both primary microglia and BV2 cells showed an enhanced release of most selected proteins but distinct secretion profiles, reflected by different clusters in the secretion heat map (Fig. 6b). Therefore, the data demonstrate that both primary microglia and BV2 cells could respond to pro-inflammatory insult LPS, but differed in their transcription and secretion profiles of inflammatory related factors, which indicates different sensitivities between these two cell types upon inflammatory stimuli.

### Difference in TGFβ signaling between primary microglia and BV2 cells

**Fig. 7:**
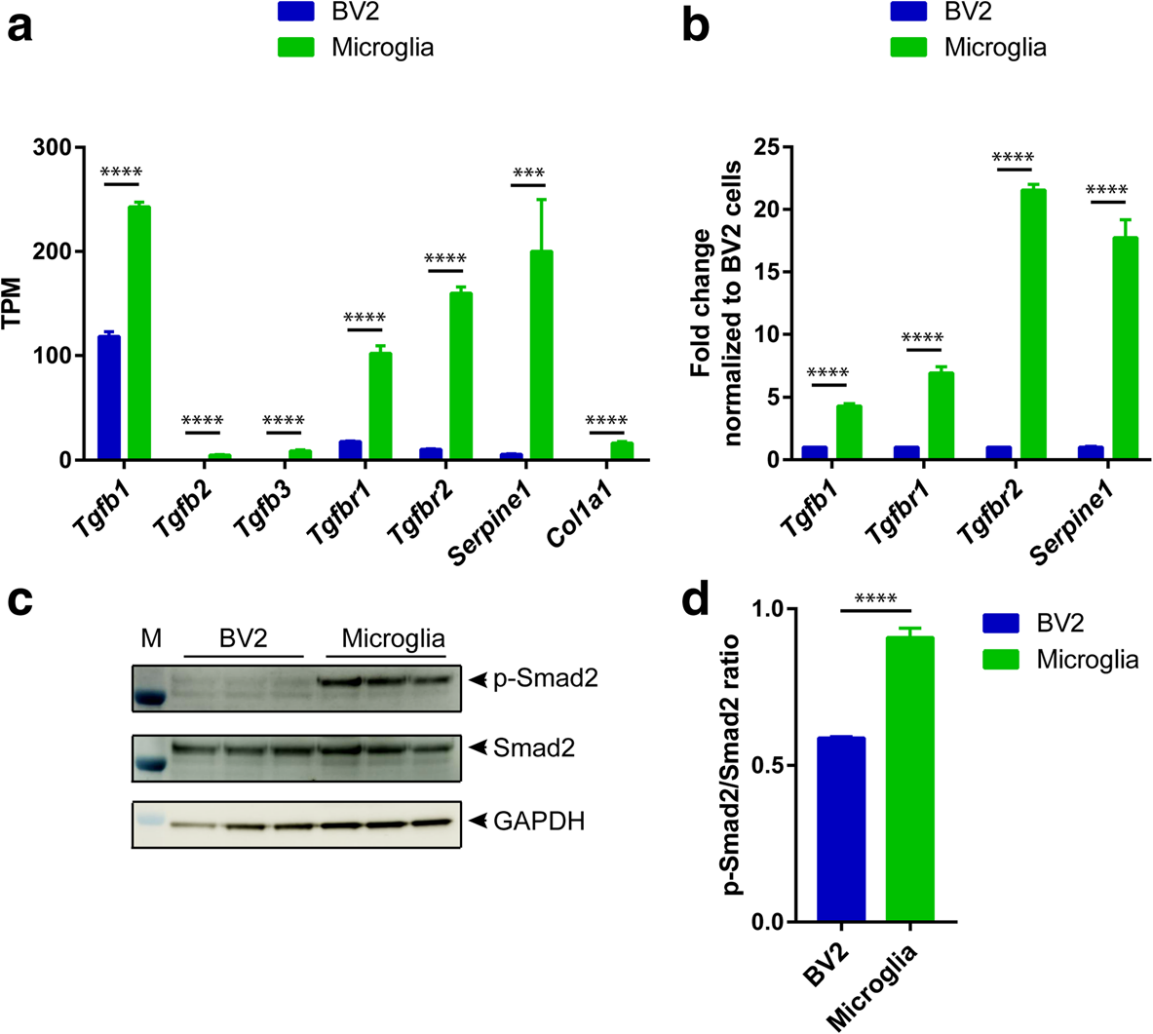
TGFβ signaling in primary microglia and BV2 cells. **a** Transcriptional expression of TGFβ signaling-related genes in primary microglia and BV2 cells by RNA-Seq. Data are shown as mean + SD, *n* = 4. **b** Transcriptional expression of TGFβ signaling-related genes in primary microglia and BV2 cells by quantitative RT-PCR. Data were normalized to the expression level in BV2 cells. Data are shown as mean + SD of triplicates. Experiment repeated twice independently. **c** Western blots were probed with the antibodies against p-Smad2, Smad2, and GAPDH. **d** Quantification of p-Smad2 signal intensity normalized to Smad2. Data are shown as mean + SD. Unpaired *t* test. ***P < 0.001; ****P < 0.0001

RNA-Seq analysis indicated that there was a difference in TGFβ signaling between primary microglia and BV2 cells, which is in agreement with Butovsky’s findings [17]. The TGFβ family includes TGFβ1, TGFβ2, and TGFβ3, which binds the receptors TGFβRII and TGFβRI to phosphorylate downstream Smad2/3 and regulate the transcription of PAI-1 and Col1a1 [25]. RNA-Seq data showed that primary microglia presented greater expression of TGFβ signaling-related genes such as *Tgfb1*, *Tgfb2*, *Tgfb3*, *Tgfbr1*, *Tgfbr2*, *Serpine1*, and *Col1a1* than BV2 cells (Fig. 7a), which was confirmed by quantitative RT-PCR (Fig. 7b). Of note, the levels of *Tgfb2*, *Tgfb3*, and *Col1a1* were too low to be detected in BV2 cells by quantitative RT-PCR. To monitor the pathway, we detected the level of p-Smad2 by Western blot. Interestingly, under basal conditions, a higher level of phosphorylated Smad2 was seen in primary microglia than in BV2 cells (Fig. 7c, d), demonstrating an increased TGFβ signaling in primary microglia. Since TGFβ signaling is critical to keep microglia quiescent, the results would be an explanation for the lower secretion levels of inflammation-related factors in primary microglia than in BV2 cells (Fig. 6b). These data supported that TGFβ signaling may be one of the key cascades to discriminate primary microglia and BV2 cells.

### Chemotactic difference between primary microglia and BV2 cells

**Fig. 8:**
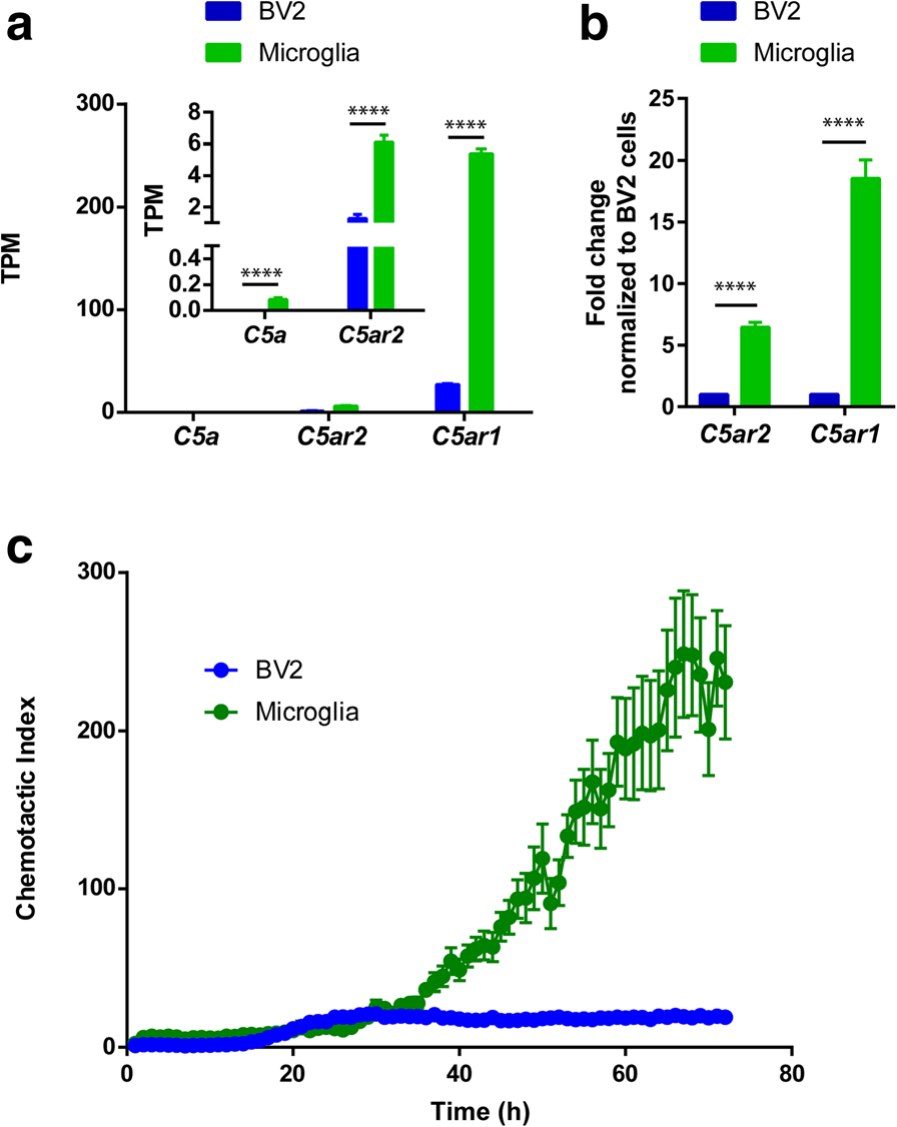
Chemotaxis comparison between primary microglia and BV2 cells. **a** Transcriptional expression of *C5a*, *C5ar1*, and *C5ar2* in primary microglia and BV2 cells by RNA-Seq. Inserted scheme depicting the expression of *C5a* and *C5ar2*. Data are shown as mean + SD, *n* = 4. Unpaired *t* test, ****P < 0.0001. **b** Transcriptional expression of *C5ar1* and *C5ar2* in primary microglia and BV2 cells by quantitative RT-PCR. Data are shown as mean + SD of triplicates. Unpaired *t* test, ****P < 0.0001. **c** Time-dependent chemotaxis of primary microglia and BV2 cells upon 11 nM C5a monitored by IncuCyte. Chemotactic index was obtained by normalization to random migration. Data are shown as mean + SD, *n* = 4. Two-way ANOVA, *P*(time) < 0.0001; *P*(cell type) < 0.0001; *P*(interaction) < 0.0001

Microglia exhibit a directional migration toward the site of injury guided by chemoattractants under pathological conditions. C5a, one of the chemoattractants, has been documented to trigger microglia direct migration through binding to its receptors C5aR1 and C5aR2 to activate downstream Rac1 signaling pathway [26, 27]. The transcriptional profile analysis revealed chemotactic difference between primary microglia and BV2 cells; we therefore sought to verify the hypothesis by chemotaxis assay using C5a as a chemoattractant. Prior to the assay, we compared expression levels of C5a and its receptors in primary microglia and BV2 cells. The RNA-Seq data revealed that primary microglia displayed higher expression levels of *C5a*, *C5ar1*, and C*5ar2* than BV2 cells (Fig. 8a), which was further confirmed by quantitative RT-PCR (Fig. 8b). We then measured chemotactic migration of the two cell types in response to 11 nM C5a by IncuCyte Zoom, a time-lapse live-cell image analysis system. The results showed an enhanced migratory activity in primary microglia compared to BV2 cells, which was reflected by the chemotactic index upon C5a, occurring around 35 h and lasting till 72 h (Fig. 8c). Therefore, we proved that primary microglia have higher chemotactic motility than BV2 cells upon C5a and thus represent a more sensitive in vitro model for chemotaxis study.

## Discussion

In this study, we presented an RNA-Seq transcriptome dataset of various microglial in vitro models, including primary microglia isolated by different methods and immortalized BV2 microglia cell line. Furthermore, based on the analysis of transcriptional differences, we compared cellular functions between primary microglia and BV2 cells by measuring LPS responses, TGFβ signaling, and chemotactic capability in parallel. The major findings are the following: (1) the CD11b MACS method was the most reliable and consistent method, which could keep the isolated microglia in a relatively quiescent state; (2) despite distinct transcriptional signature, BV2 cells shared most immune functions, including the responses to LPS with primary microglia, but showed differences in TGFβ signaling and chemotaxis. Hence, our study characterized the usefulness and limitations for certain microglial isolation methods and BV2 cells and provides valuable insights into the selection of proper microglia as in vitro models for specific investigation.

Our current study focused on microglial isolation from postnatal mouse brains with a mixed glial culture system. Transcriptional differences have been reported between isolated microglia from postnatal and adult brains [17]. Plus, environmental factors including culture conditions impact microglial transcriptome [28], which brings into concern the application of microglial culture. Nevertheless, postnatal microglia isolation and culture is still a useful tool for microglial studies due to high yield and relatively easy manipulation and culture. Most importantly, postnatal microglia could recapitulate most phenotypes of microglia in vivo, including cytokine secretion, chemotaxis, and phagocytosis [6]. Here, we compared mouse microglia from postnatal brains with three most popular isolation methods at the transcriptional level and discovered that cells isolated from CD11b MACS and shaking methods were in a relatively resting state, as compared to those from mild trypsinization isolation. This was evidenced not only by microglial activation genes but also by microglial quiescent genes such as TGFβ signaling-related genes. These findings suggest that CD11b MACS- and shaking-isolated microglia are more suitable for comparison of gene expression profiles and functions when treated by potential therapeutic interventions. However, we could not exclude that subtle differences in purity and subpopulation of isolated microglia from distinct methods may influence the results. Our data are in accordance with previous reports of unwanted transcriptional changes and activation of microglia upon enzymatic digestion such as trypsin [29]. Moreover, differential adherence-based isolation such as shaking [30] and mild trypsinization [13] is difficult to control and reproduce. Shaking speed and duration, as well as trypsinization time, depend on microglial confluence in the mixed glial culture and thus differ batch to batch, which may explain the contradiction between our study and Lin et al.’s study [31]. In contrast, CD11b MACS method relies on antigen–antibody interaction [12], which is comparatively consistent and reproducible. Furthermore, CD11b MACS method allows co-harvesting of astrocytes and microglia with high purity from the same mixed culture by depletion or positive selection of microglia from MACS column. Therefore, based on our culture experience and RNA-Seq data, CD11b MACS method is considered an efficient and consistent method to isolate pure and inactive microglia, which can be routinely used for mechanistic studies and compound screening targeting microglial functions.

Due to limited yield of primary microglia produced from mouse brains, a BV2 cell line is frequently used as an alternative owing to a shorter preparation time and its homogeneous population across experiments. However, the validation of BV2 cells as a sufficient substitute for primary microglia has been debated [17]. At present, we performed RNA-Seq on primary microglia and BV2 cells under non-treated conditions and compared their biological pathways and cellular functions. In addition to common properties of immortalized cell lines (e.g., increased proliferation and adherence), BV2 cells retain most crucial functions of microglia in immune response and inflammation. For example, BV2 cells responded to LPS as primary microglia with enhanced transcript expression and secretion but had distinct transcription and secretion profiles within our selected panel. This finding further supported previous observation that although both BV2 cells and primary microglia express Iba1, a microglia marker, BV2 cells exhibit far less induction of some pro-inflammatory genes and much lower cytokine secretion levels in response to LPS, when compared with primary microglia [15, 16]. Furthermore, some specific signal pathways and functions, such as TGFβ signaling and chemotaxis upon C5a, substantially differ between primary microglia and BV2 cells from transcriptional and functional aspects. These results characterized the usefulness and limitations of BV2 as an alternative in vitro model. Hence, our analysis raised concerns about appropriate cell models when performing a microglial study to address specific immunological and inflammatory responses.

There are a few directions for future investigation. First, the primary microglia in our study are of postnatal origin. Additional genome-wide transcriptional and functional studies should be performed for direct comparison with microglia from adult tissues. Second, this study only looks into intervention-free situations. Further studies are required to investigate transcriptional changes upon different stimuli. Third, although rodent models are effective systems to investigate the emerging functions of microglia, further research work should be carried out to compare rodent and human microglia at the transcription and function levels to facilitate the translation from preclinical to human studies.

## Conclusions

The study provides a systematic comparison of primary microglia isolated by different methods generally used in the literatures and with BV2 cells. Data presented here highlight the following important points: (1) cell isolation procedure and culture conditions significantly influence the transcriptional expression and activation states of microglia; (2) caution has to be taken in using BV2 cells as a microglial surrogate. Our data provide a complement to current microglial research and valuable insights into the selection of appropriate microglia as in vitro models under certain circumstances.

## Additional files

Additional file 1: Characterization of isolated microglia. **a** Purity of isolated microglia from the three isolation methods. Purity was quantified by counting Iba1^+^ microglia in total cells, which were indicated by DAPI and immunocytochemical staining from at least five randomly selected fields. **b** Representative astrocyte images showing positive GFAP staining (Iba1^+^, green; GFAP^+^, red). DAPI indicates nuclei. Scale bar, 50 μm. (TIF 856 kb)

Additional file 2: Sequencing depth of all the RNA-Seq samples. **a** Read count of all samples. **b** Mapping percentage of all samples. (TIF 447 kb)

Additional file 3: PCA plot on transcriptomes of all samples. (TIF 255 kb)

Additional file 4: Fold changes and average expression level of all transcripts among various isolation methods. (XLSX 4831 kb)

Additional file 5: Fold changes and average expression level of all transcripts among all groups in LPS, Aβ, and aging studies. (XLSX 723 kb)

## Abbreviations

C5a: Complement component 5a
LPS: Lipopolysaccharide
MACS: Antigen–antibody-mediated magnetic cell sorting
PAI-1: Plasminogen activator inhibitor-1
RNA-Seq: RNA sequencing
TGFβ: Transforming growth factor beta
