# Supplementary figures and images for: RNA sequencing analysis reveals quiescent microglia isolation methods from postnatal mouse brains and limitations of BV2 cells
Source: J Neuroinflammation. 2018 May 22;15:153. doi: 10.1186/s12974-018-1195-4 (PMC5964710; doi:10.1186/s12974-018-1195-4)

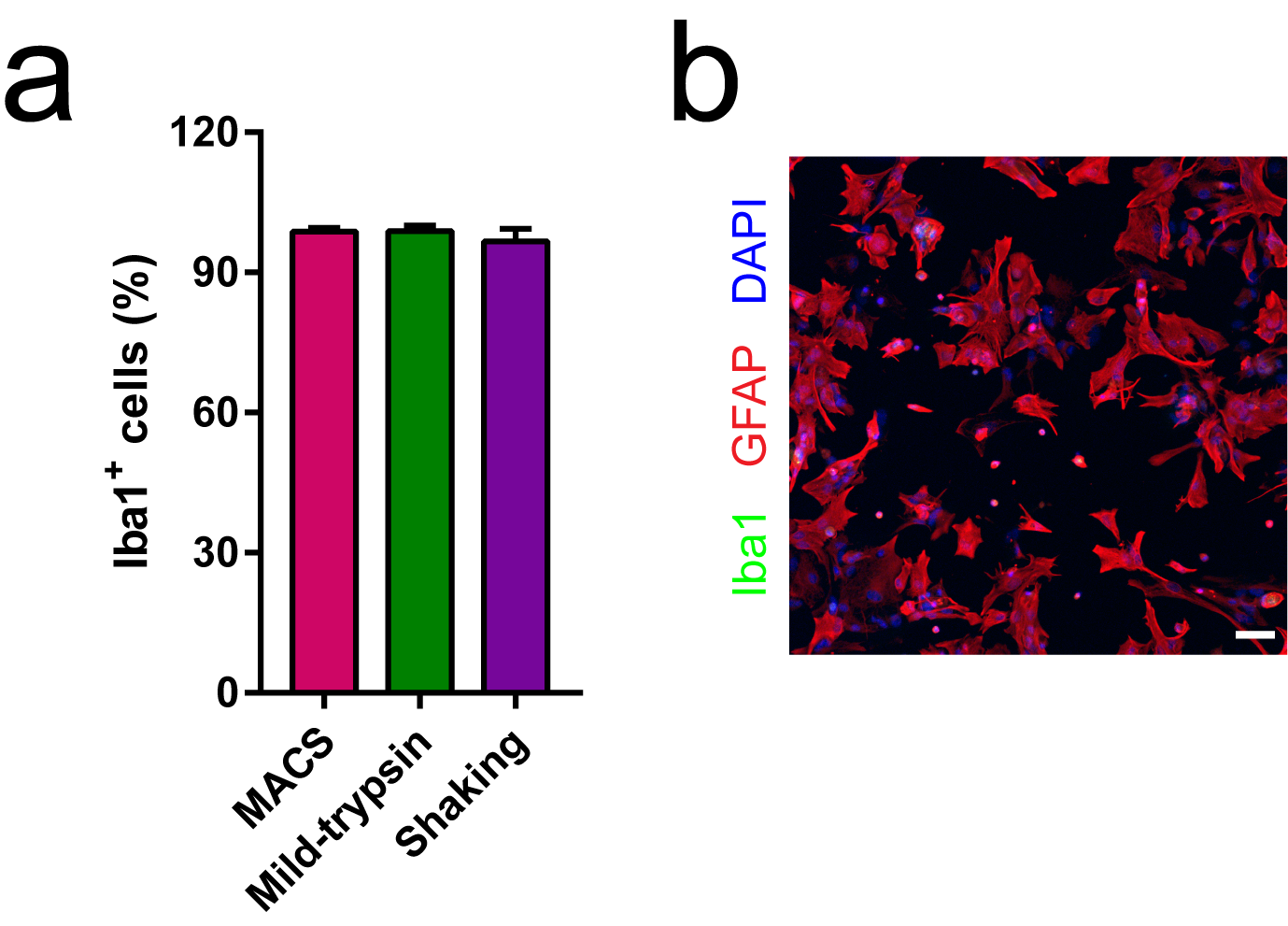

Supplement: Supplementary file 1 — Characterization of isolated microglia. a Purity of isolated microglia from the three isolation methods. Purity was quantified by counting Iba1+ microglia in total cells, which were indicated by DAPI and immunocytochemical staining from at least five randomly selected fields. b Representative astrocyte images showing positive GFAP staining (Iba1+, green; GFAP+, red). DAPI indicates nuclei. Scale bar, 50 μm. (TIF 856 kb) [file 12974_2018_1195_MOESM1_ESM.tif]

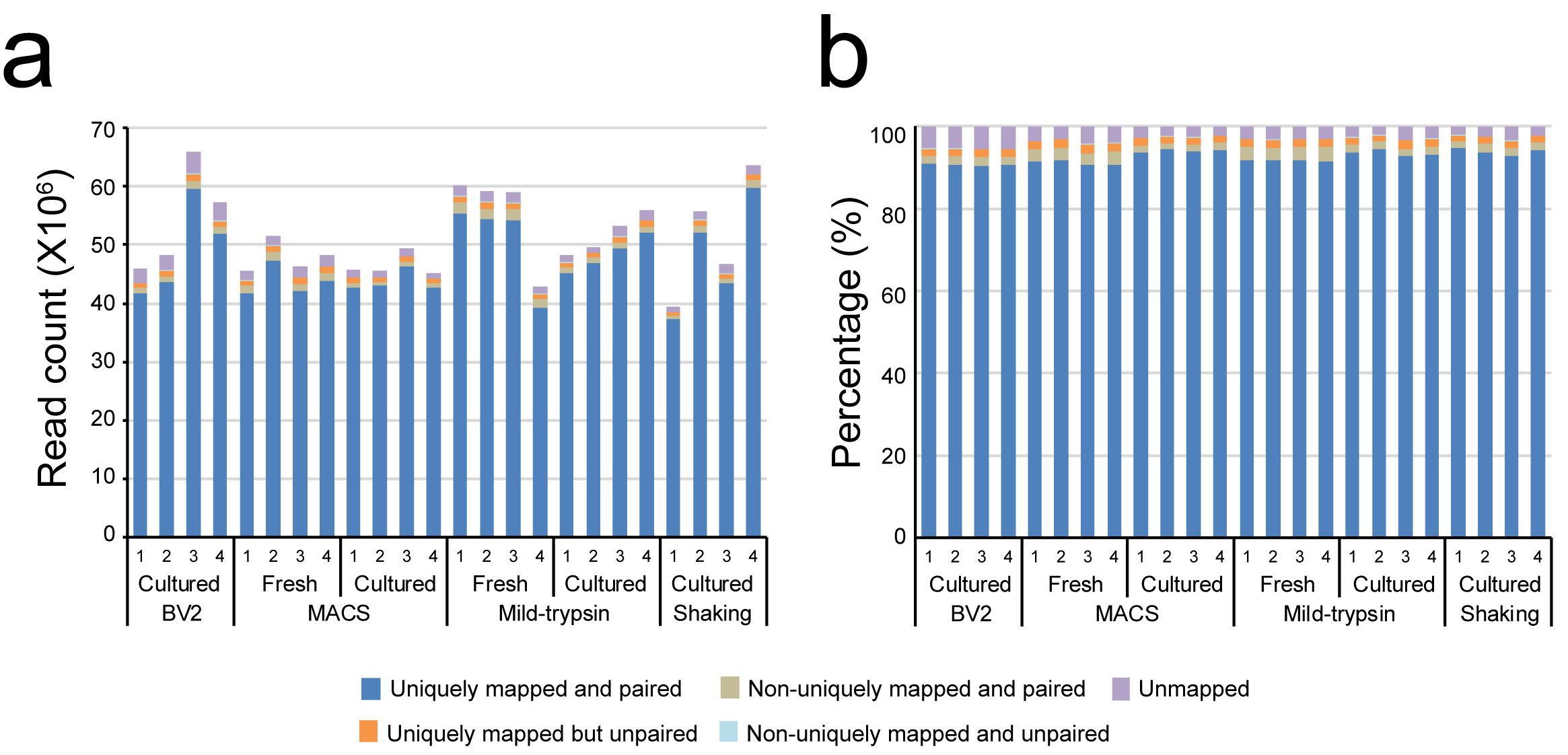

Supplement: Supplementary file 2 — Sequencing depth of all the RNA-Seq samples. a Read count of all samples. b Mapping percentage of all samples. (TIF 447 kb) [file 12974_2018_1195_MOESM2_ESM.tif]

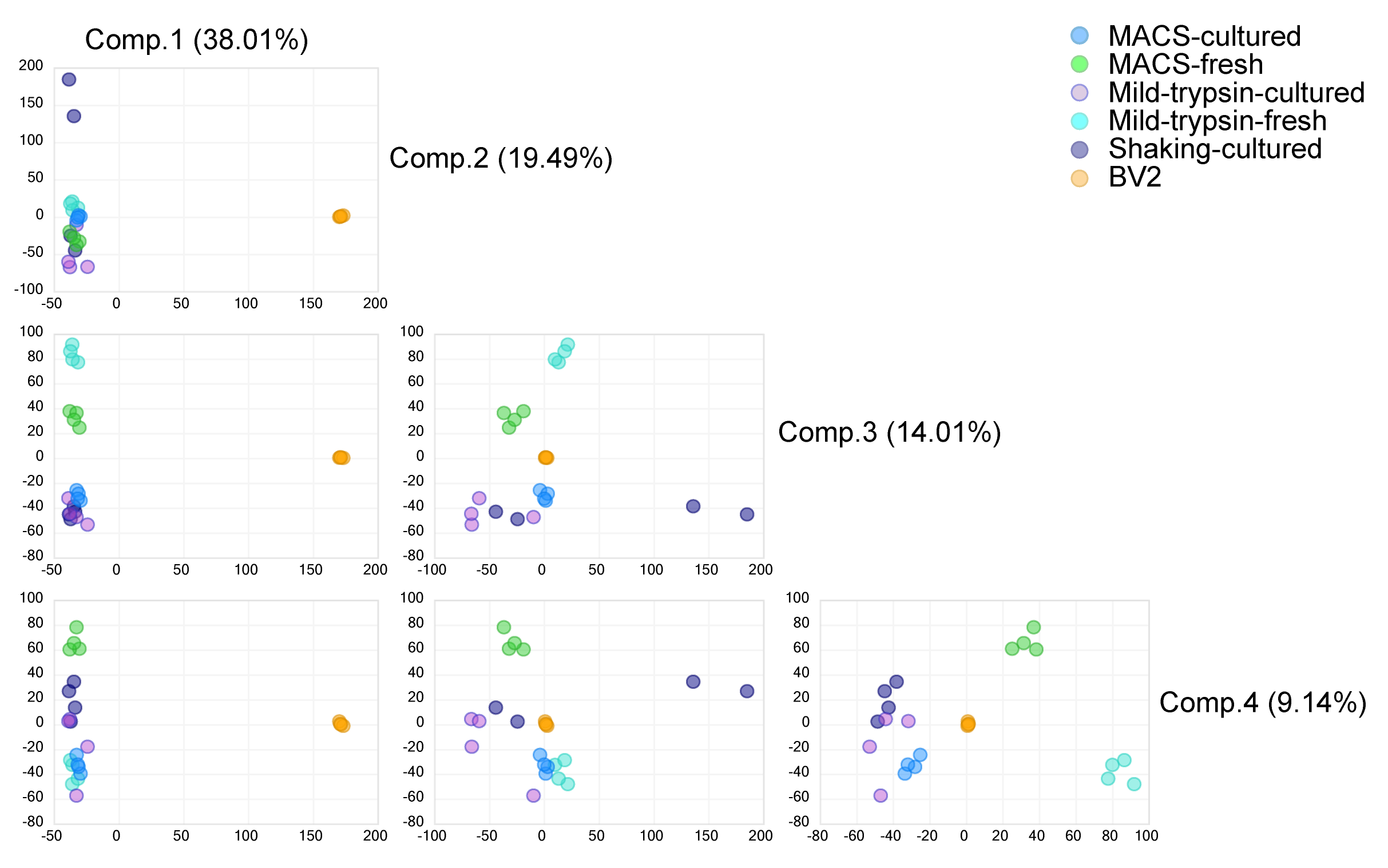

Supplement: Supplementary file 3 — PCA plot on transcriptomes of all samples. (TIF 255 kb) [file 12974_2018_1195_MOESM3_ESM.tif]
